# Middle Meningeal Artery (MMA) Embolization for Recurrent Subacute Subdural Hematoma With Complete Resolution Following Transient Radiological Size Progression: A Case Report and Literature Review

**DOI:** 10.7759/cureus.83034

**Published:** 2025-04-26

**Authors:** Farid Bouhouf, Abdullah F Alsubaii, Khalid A Alshammari, Bashaer S AlHarbi, Haya AlTamimi

**Affiliations:** 1 Department of Neurosurgery, King Abdulaziz Medical City, Ministry of National Guard Hospital, Riyadh, SAU; 2 College of Medicine, King Saud Bin Abdulaziz University for Health Sciences, Riyadh, SAU; 3 Department of Neurosurgery, National Neuroscience Institute, King Fahad Medical City, Riyadh, SAU

**Keywords:** case report, literature review, middle meningeal artery embolization, recurrent subdural hematoma, subdural hematoma

## Abstract

Recurrent subdural hematoma (RSDH) is a condition characterized by repeated blood accumulation beneath the dura mater, which often poses challenges in clinical management. Conventional surgical interventions, such as burr hole irrigation and drainage, are standard treatments but carry a risk of recurrence. Therefore, middle meningeal artery (MMA) embolization has recently emerged as a promising alternative to address RSDH.

This case report provides an overview of an RSDH in a 56-year-old female who underwent successful MMA embolization. She initially experienced a worsening of the hematoma with an acute-on-chronic component, which later regressed significantly, leading to near-complete resolution over a one-year follow-up period. Such an outcome has not been well documented in the existing literature and therefore represents an important case study that underscores the potential of MMA embolization as a viable option for RSDH, even in cases of transient post-procedural radiological hematoma expansion.

The findings emphasize the importance of careful patient monitoring and suggest that MMA embolization may offer an effective, minimally invasive alternative to surgery in select cases. It also highlights the need for ongoing research to determine the long-term effectiveness and safety of the procedure.

## Introduction

Recurrent subdural hematoma (RSDH) is a medical condition in which blood repeatedly accumulates under the layers of the dura mater following surgical evacuation [1]. This situation poses a challenge in terms of clinical management and often requires immediate and targeted intervention. Typically, burr hole irrigation and drainage or a mini-craniotomy are performed to treat a subdural hematoma when surgically indicated. However, there are numerous cases where recurrences of SDH have been reported [1,2]. In one systematic review, the rate of recurrence was estimated to be 12.8% [3]. The underlying mechanisms of RSDH are multifactorial, primarily involving incomplete evacuation of loculated hematomas, rebleeding from fragile neomembranes (which contain leaky, immature blood vessels), and poor brain re-expansion, particularly in elderly patients with cerebral atrophy. Additional contributors include coagulopathies (whether medication-induced or intrinsic) and technical limitations of surgical drainage, such as inadequate irrigation or suboptimal drain placement. Together, these factors create conditions that favor recurrent hemorrhage and impede complete hematoma resolution [4,5]. Recently, it has been suggested to use embolization of the middle meningeal artery (MMA) rather than surgery to address RSDH [6,7]. Some case reports have demonstrated its effectiveness in this regard [8-10]. In this case report, we aim to provide a comprehensive overview of a patient with RSDH who underwent successful MMA embolization, during which she initially experienced a radiological progression in hematoma size, followed by near-complete resolution, as confirmed by serial brain CT scans over a one-year follow-up period.

## Case presentation

**Figure 1:**
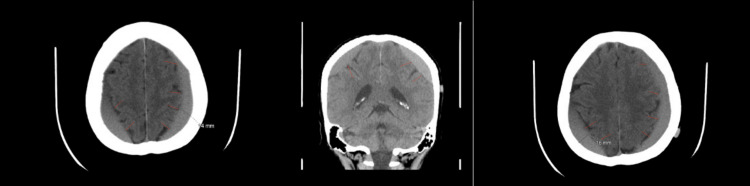
Brain CT pre-burr hole evacuation. There is an interval increase in the size and density of bilateral subdural hemorrhages, with minimal associated mass effect. The left-sided subdural hemorrhage shows a maximum thickness of 1.4 cm, compared to the previous measurement of 0.9 cm. The right-sided subdural hematoma shows a maximum thickness of 1.6 cm, compared to the previous measurement of 0.7 cm.

A 56-year-old woman with a known history of hypothyroidism and hypertension (HTN) presented with a one-month history of headache and multiple episodes of vomiting following a fall-related head injury. Initially, she was diagnosed with an SDH at a different hospital and was managed conservatively until her symptoms worsened, accompanied by radiographic progression of the hematoma on CT imaging (Figure 1). The patient was subsequently admitted to our hospital as a case of bilateral chronic SDH and underwent surgical evacuation via a left parietal burr hole and two right frontal and parietal burr holes, followed by irrigation, drainage, and insertion of a right frontal drain. Intraoperatively, dark, pressurized subdural fluid was observed.

**Figure 2:**
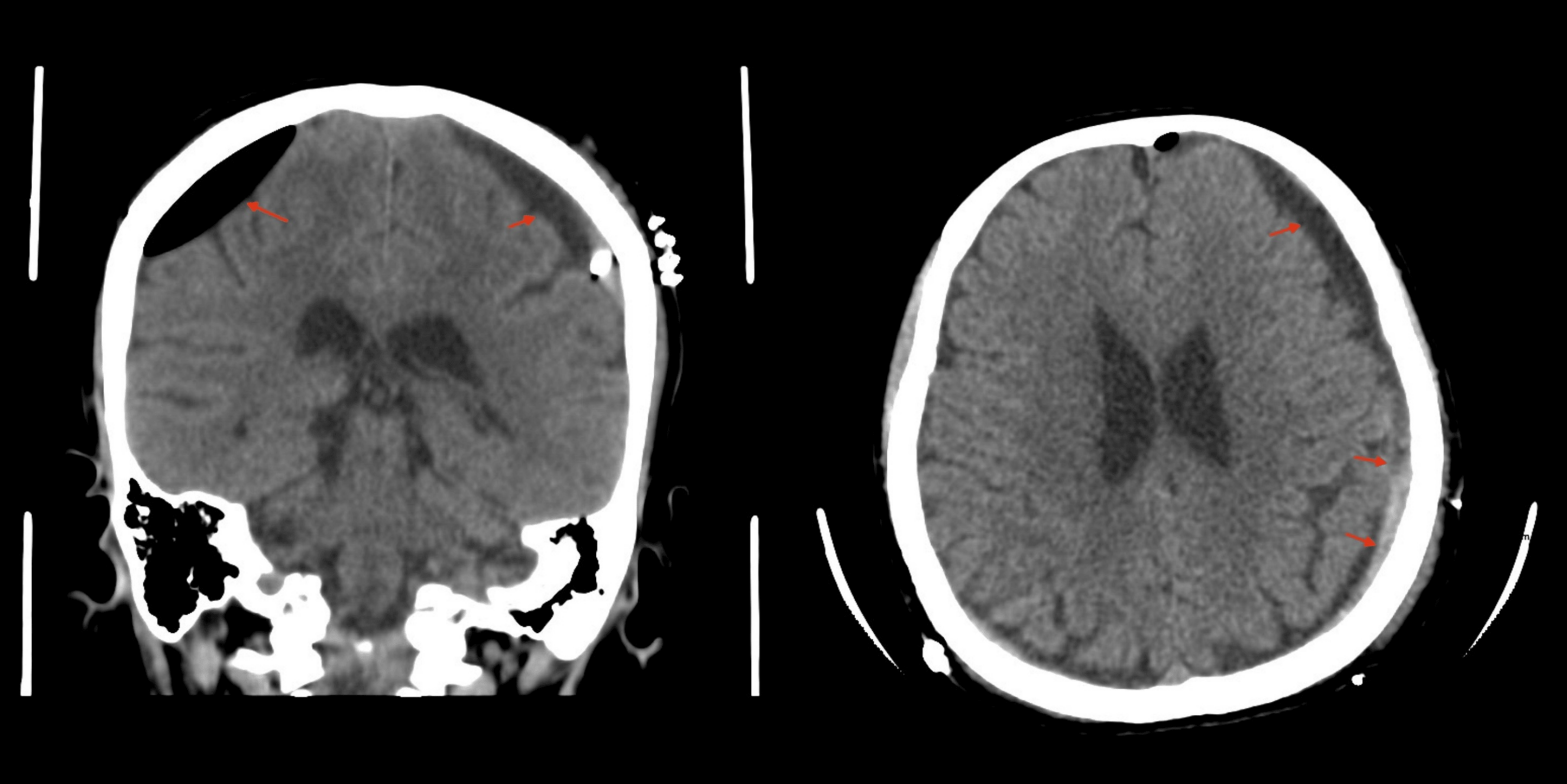
Brain CT post-burr hole evacuation. Postoperative axial and coronal brain CT scans showing subdural hemorrhage with a maximum thickness of 0.4 cm on the left side and 0.7 cm on the right side.

**Figure 3:**
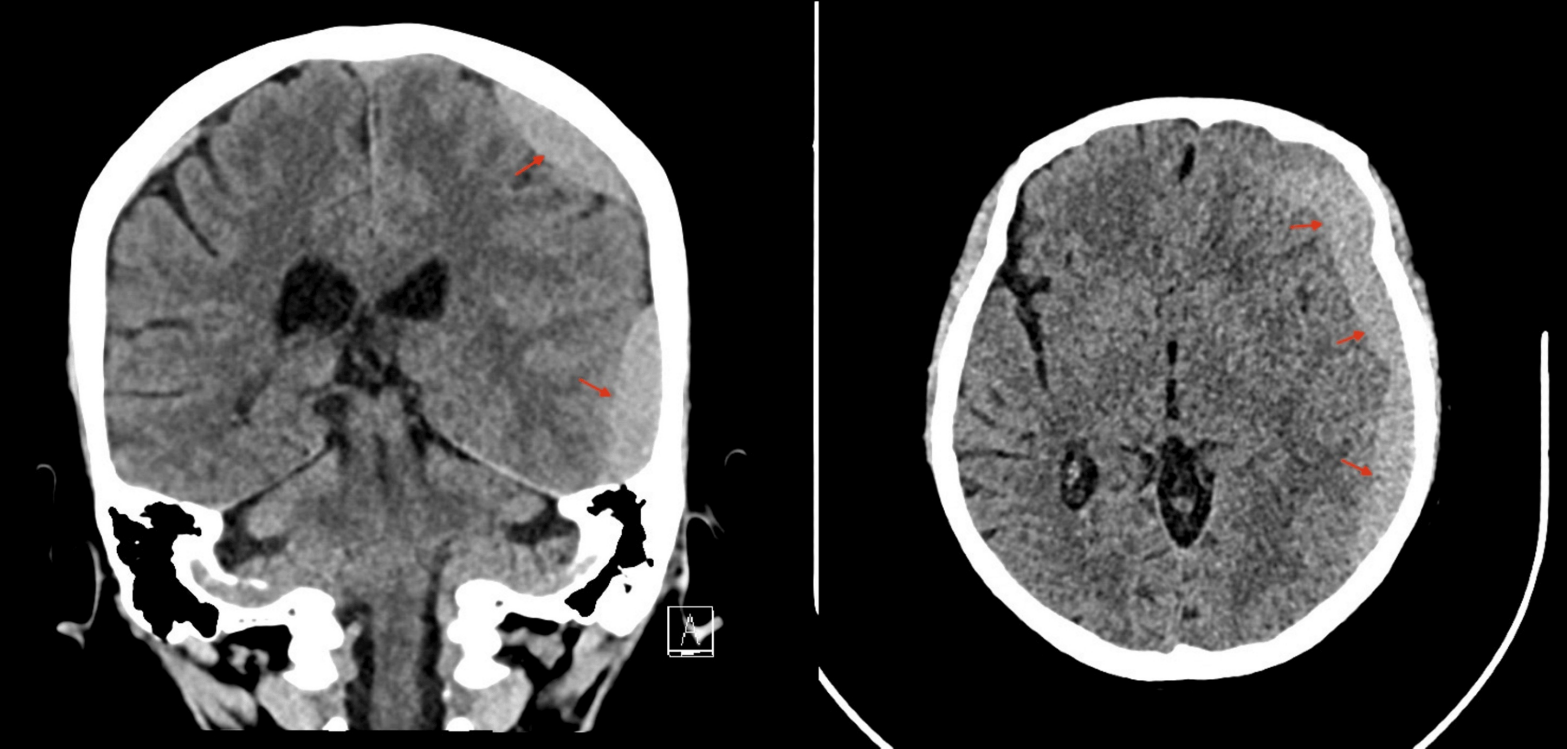
Second admission brain CT pre-MMA embolization. Axial and coronal brain CT scans showing an interval increase in the size of the left cerebral convexity subdural hematoma, with a maximum thickness of 1.7 cm compared to the previous 1 cm. Interval development of a minimal midline shift toward the right side, measuring 0.4 cm. MMA: Middle meningeal artery.

**Figure 4:**
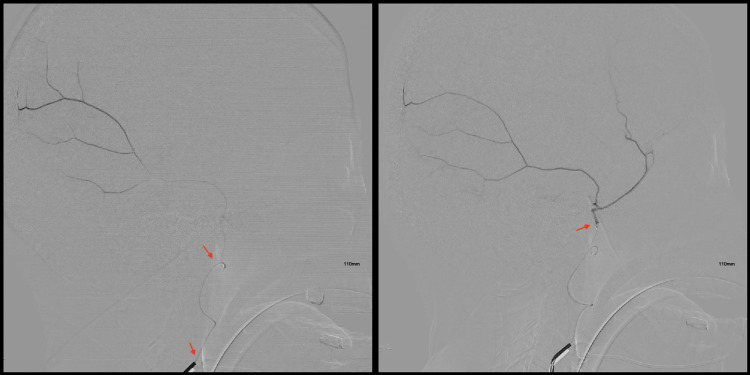
MMA embolization. The left MMA was selectively catheterized using a technique with SONIC. The microcatheterization was performed in two embolization steps: first, catheterization of the anterior frontal branch of the MMA; second, catheterization of the posterior parietal branch of the MMA, followed by Onyx embolization. MMA: Middle meningeal artery.

**Figure 5:**
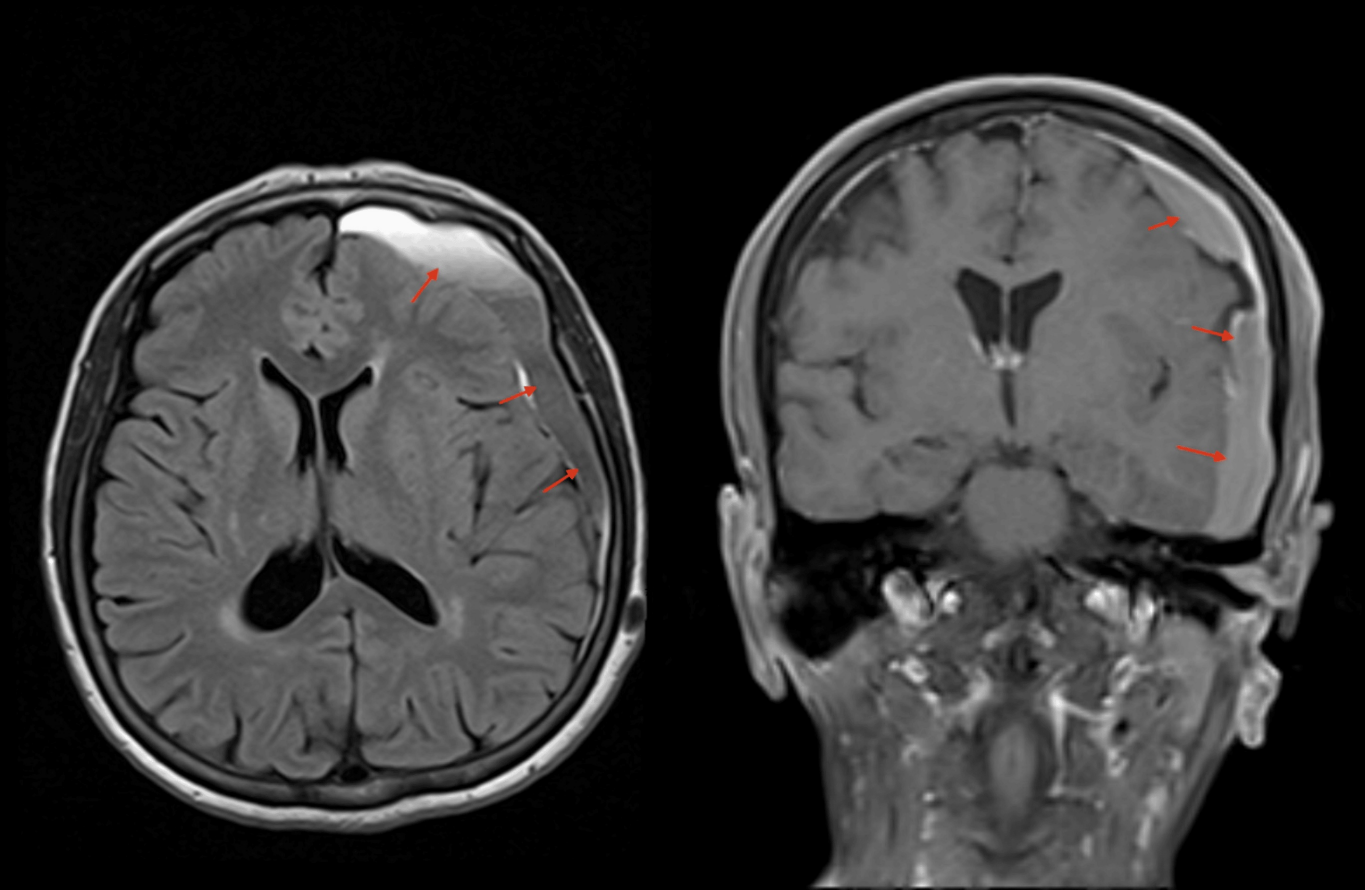
Brain MRI post-MMA embolization. Follow-up axial and coronal brain MRI demonstrates bilateral cerebral convexity subdural hematomas with interval stability in terms of maximum thickness and midline shift. MMA: Middle meningeal artery.

Postoperatively, the patient showed significant clinical improvement, with follow-up CT imaging revealing decreased SDH volume: a 0.4 cm residual hemorrhage on the left and 0.7 cm on the right (Figure 2). One month later, during her follow-up, she returned with a severe headache, without a history of trauma. Repeated CT imaging (Figure 3) demonstrated recurrence and progression of SDH with acute-on-chronic hemorrhage. Middle meningeal artery (MMA) embolization was performed (Figure 4). After the procedure, she recovered well and was managed medically for headache. A comprehensive workup was performed to rule out coagulopathy as well as vascular malformations and fistulas through MRA and MRV. Postoperative MRI confirmed stability in terms of thickness and midline shift of the bilateral subdural hematoma (Figure 5).

**Figure 6:**
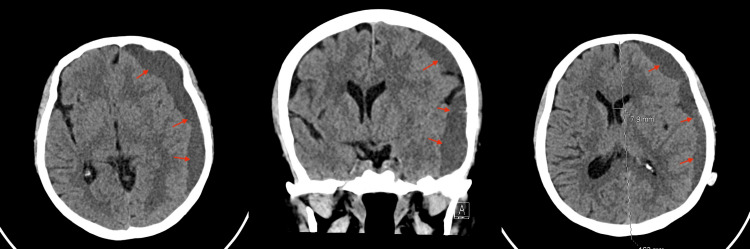
A total of 18-day brain CT post-MMA embolization. There is an interval increase in the size of the left cerebral convexity subdural hematoma. The maximum thickness measures approximately 2 cm, causing a mass effect, more prominent on the left temporal lobes. There is also an interval worsening of the midline shift to the right side, now measuring approximately 8 mm. MMA: Middle meningeal artery.

**Figure 7:**
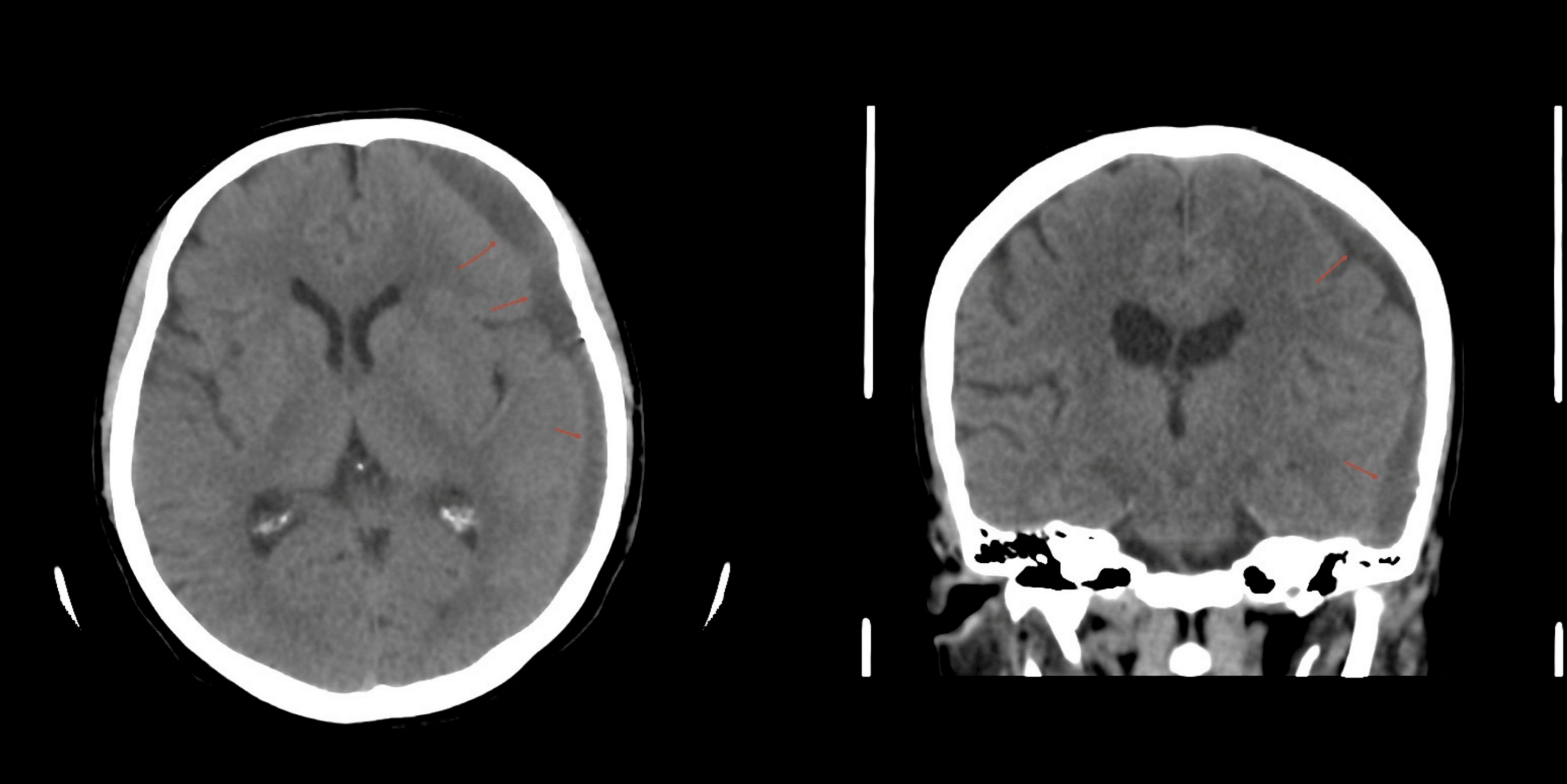
One-month post-MMA embolization. Interval decrease in the size of the left cerebral convexity subdural hematoma, with a maximum thickness of 1.5 cm compared to the previous 2 cm, associated with improvement of the rightward midline shift, now measuring 4.8 mm. MMA: Middle meningeal artery.

**Figure 8:**
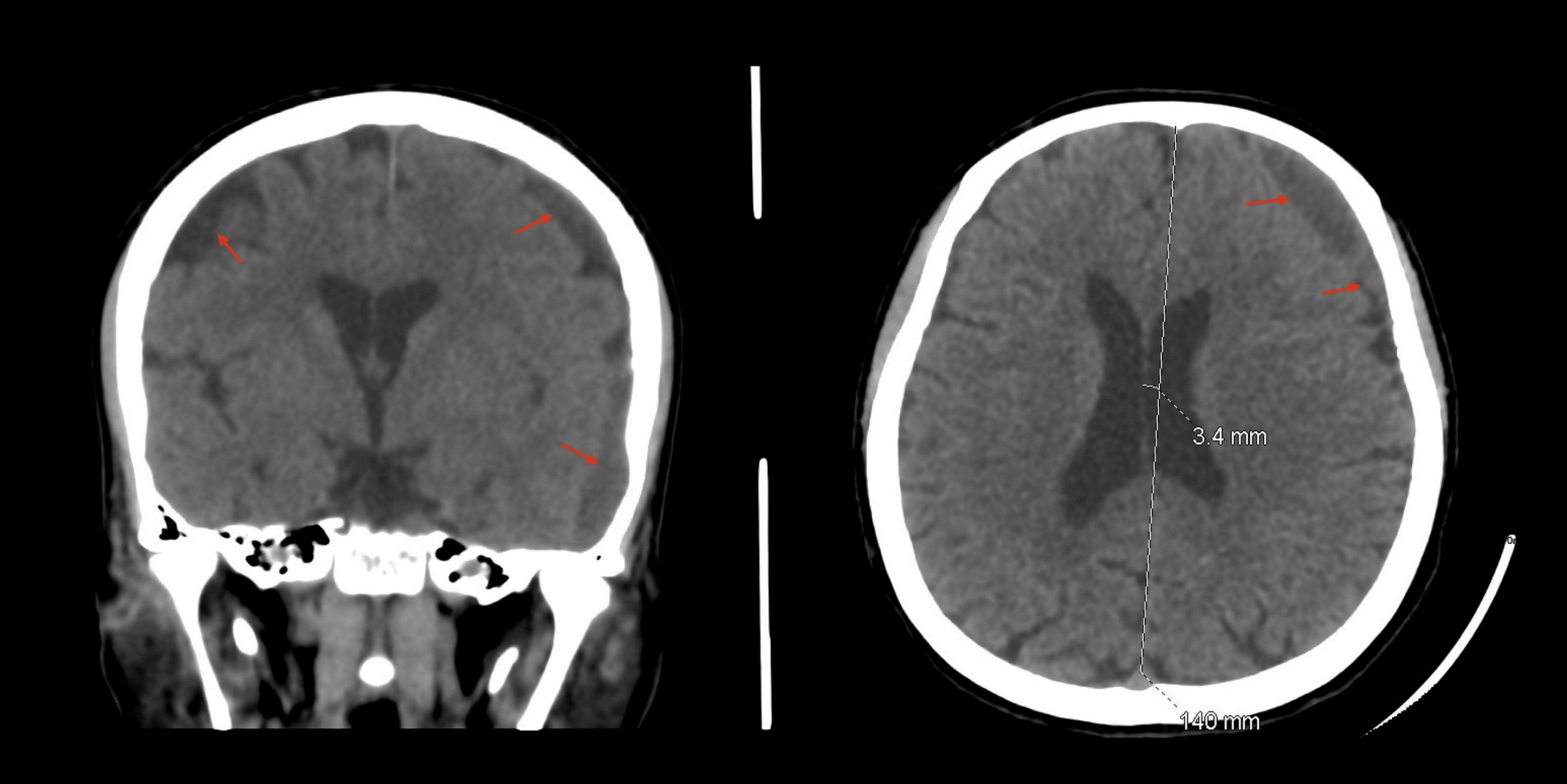
Six weeks post-MMA embolization. Evolutionary changes with a mild interval decrease in the size of the left cerebral convexity subdural hematoma, with a midline shift of 3.4 mm and a maximum thickness of 1.1 cm compared to the previous 1.5 cm. MMA: Middle meningeal artery.

**Figure 9:**
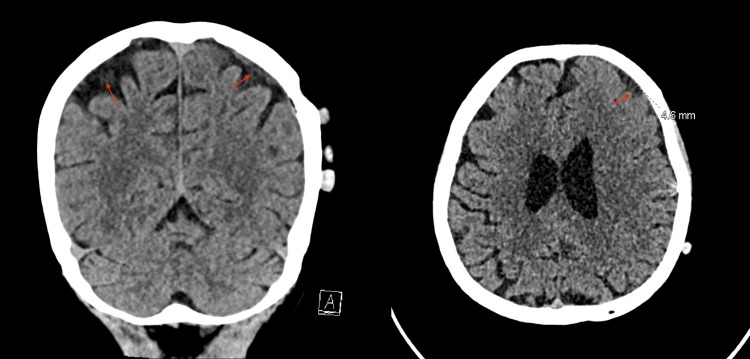
Eight weeks post-MMA embolization. Axial and coronal brain CT scans showing significant interval resolution of the subdural hemorrhage. MMA: Middle meningeal artery.

The patient was stable and kept under observation with serial CT images, which eventually revealed a worsening in the size of the hematoma (8 mm midline shift) with mixed isodense and hypodense liquefaction 18 days post-embolization, despite no traumatic or hematologic cause being identified (Figure 6). Nevertheless, the patient remained clinically stable and fully conscious but continued to complain of headaches despite medical treatment. A decision for surgical reintervention was discussed with the patient, including the risks and benefits, but she declined. Close observation continued with no concerns regarding her clinical status. Thirty days post-embolization, a serial CT image revealed sufficient improvement in the size of the hematoma (Figures 7-8). The patient was then discharged and seen at follow-up visits. One month later, a repeated outpatient brain CT scan revealed significant interval resolution of the subdural hematoma (Figure 9). At her one-year follow-up, a CT scan confirmed no recurrence of SDH with almost full resolution. Throughout all time periods, the patient remained clinically stable with no neurological deficit. 

## Discussion

Subdural hematomas (SDHs) are classified according to their chronology: SDHs developing within less than one day are considered hyperacute, with isodense liquefaction; those occurring between 1 and 3 days are classified as acute, with hyperdense liquefaction [11]. SDHs persisting beyond 3 days are categorized as subacute, where the collection becomes isodense, or chronic, where the collection becomes hypodense. A subacute SDH (saSDH) develops from an acute SDH within 4 to 21 days of a head injury; nevertheless, the distinction between saSDHs and chronic SDHs remains uncertain. As a result, saSDHs are sometimes classified as chronic SDHs (CSDH) [12].

Treatment options vary according to the symptomatology and degree of the hematoma. Burr hole evacuation and drainage, per medical literature, are recommended over mini-craniectomy or other options for patients who are radiologically and clinically indicated for surgery [13]. Nonetheless, traditional methods face challenges with SDH recurrence. According to one study, surgical drainage of CSDH fails to cure 9.4-30% of patients [11]. Meanwhile, other studies have found recurrence rates to vary widely, from 2% to 37% [14].

Some patients undergo repeated surgical treatment; however, if the initial surgical treatment fails once, further recurrences become more likely, with rates estimated as high as 46% [7]. Risk factors associated with an increased recurrence rate include patient-related factors (e.g., alcohol abuse, anticoagulant and/or antiplatelet use, brain atrophy, bleeding tendency, or intracranial hypotension) and radiologic features (e.g., hematoma location, volume, and density). Age, sex, comorbidities, and the cause of the hematoma do not significantly impact recurrence rate [14].

Several studies highlight MMA embolization as an alternative treatment option for recurrent SDH. The rationale involves reducing blood supply to the subdural membranes enough so that there is a shift from continuous leakage and accumulation of blood products toward reabsorption. Moreover, it is believed that chronic SDH is primarily caused by repeated small-scale bleeding into the subdural collection from delicate new blood vessels within the SDH membrane, which arise from distal branches of the MMA. As a result, it could potentially stop the ongoing bleeding [4].

Therefore, MMA embolization is increasingly advocated for recurrent SDH. A meta-analysis study supports MMA embolization’s safety and efficacy. A review of 35 studies (746 patients) reported a low complication rate of 3.9-8.9% and a significant reduction in recurrence rate. The study suggests that using MMA embolization as an alternative option can be beneficial in terms of preventing recurrences and reducing complications associated with surgical procedures [15].

Tempaku analyzed 13 patients from 110 recurrent CSDH cases post-burr hole evacuation. These 13 experienced hematoma recurrences and required additional surgical treatment. Five patients underwent MMA embolization, and none experienced further SDH recurrence during a follow-up period ranging from 4 to 60 weeks [7]. Similarly, Link TW examined the effects of MMA embolization in 60 CSDH patients and observed an 8.9% failure rate and a 68.9% improvement rate (≥50% reduction) [16]. However, the failure rate for recurrent SDH (RSDH) remains poorly defined.

Our patient was initially operated on for bilateral CSDH but later developed a gradual progression of acute bleeding on top of chronic hematoma, as documented in serial CT scans, with no identifiable associated risk factors. MMA embolization was pursued as an alternative treatment option. At 18 days post-MMA embolization, imaging showed radiological worsening (midline shift of 8 mm). Nevertheless, a reduction eventually began to appear. Interestingly, as the patient declined further surgery, we were able to observe the natural evolution of the hematoma size and chronology. We noted that after MMA embolization, the hematoma could initially become complicated and increase in size for up to 18 days; however, in clinically stable patients, the hematoma may begin to regress without additional intervention.

Thus, we hypothesize that post-embolization temporary worsening may reflect a chronological phase transition (from subacute to chronic) accompanied by density changes, followed by spontaneous regression and near-complete resolution. Prior studies report <2% complication rates and significant improvements post-embolization, but none have documented interval hematoma expansion. Moreover, patients generally improved significantly following embolization, with a substantially reduced recurrence rate [17,18].

Our findings emphasize the potential for temporary radiological progression post-embolization, highlighting the importance of closely monitoring patients, even if hematoma size increases, provided the patient remains clinically stable. However, it is crucial to note that allowing a hematoma to grow unchecked could have catastrophic consequences, and surgical intervention may eventually be warranted [19,20].

## Conclusions

While traditional surgical evacuation remains the standard treatment for RSDH, MMA embolization has emerged as a promising and effective alternative. Notably, even in the context of transient post-procedural hematoma expansion, clinically stable patients may be safely managed with close observation rather than immediate re-intervention, a strategy that warrants validation through prospective studies. The successful outcome in this case, characterized by delayed hematoma resolution despite initial expansion, challenges conventional management paradigms and highlights two critical neurosurgical principles: first, radiologic progression does not invariably mandate immediate re-intervention in neurologically intact patients, suggesting that careful observation may be appropriate in select cases; second, the natural history of post-embolization SDH may include a transitional phase with temporary increases in hematoma size and density prior to resorption. Our case report underscores the potential of focused interventions in neurosurgical practice and emphasizes the importance of ongoing research to determine the long-term effectiveness and safety of such procedures. As the domain of neurosurgery continues to advance, MMA embolization may redefine the therapeutic algorithm for RSDH, offering a safer alternative to repeated craniotomies in select populations.
